# Analysis of Composition and Structure of Coastal to Mesopelagic Bacterioplankton Communities in the Northern Gulf of Mexico

**DOI:** 10.3389/fmicb.2012.00438

**Published:** 2013-01-18

**Authors:** Gary M. King, Conor B. Smith, Bradley Tolar, James T. Hollibaugh

**Affiliations:** ^1^Department of Biological Sciences, Louisiana State UniversityBaton Rouge, LA, USA; ^2^Department of Marine Studies, University of GeorgiaAthens, GA, USA

**Keywords:** northern Gulf of Mexico, bacterioplankton, diversity, phylogenetic community structure, thaumarchaeota

## Abstract

16S rRNA gene amplicons were pyrosequenced to assess bacterioplankton community composition, diversity, and phylogenetic community structure for 17 stations in the northern Gulf of Mexico (nGoM) sampled in March 2010. Statistical analyses showed that samples from depths ≤100 m differed distinctly from deeper samples. SAR 11 α-Proteobacteria and Bacteroidetes dominated communities at depths ≤100 m, which were characterized by high α-Proteobacteria/γ-Proteobacteria ratios (α/γ > 1.7). Thaumarchaeota, Firmicutes, and δ-Proteobacteria were relatively abundant in deeper waters, and α/γ ratios were low (<1). Canonical correlation analysis indicated that δ- and γ-Proteobacteria, Thaumarchaeota, and Firmicutes correlated positively with depth; α-Proteobacteria and Bacteroidetes correlated positively with temperature and dissolved oxygen; Actinobacteria, β-Proteobacteria, and Verrucomicrobia correlated positively with a measure of suspended particles. Diversity indices did not vary with depth or other factors, which indicated that richness and evenness elements of bacterioplankton communities might develop independently of nGoM physical-chemical variables. Phylogenetic community structure as measured by the net relatedness (NRI) and nearest taxon (NTI) indices also did not vary with depth. NRI values indicated that most of the communities were comprised of OTUs more distantly related to each other in whole community comparisons than expected by chance. NTI values derived from phylogenetic distances of the closest neighbor for each OTU in a given community indicated that OTUs tended to occur in clusters to a greater extent than expected by chance. This indicates that “habitat filtering” might play an important role in nGoM bacterioplankton species assembly, and that such filtering occurs throughout the water column.

## Introduction

The northern Gulf of Mexico (nGoM) supports some of the most economically and ecologically valuable marine and coastal ecosystems in North America (Rabalais et al., 1996). A number of process-based studies (mostly within the Mississippi River plume) have analyzed bacterial numbers, respiration rates, production rates, and nitrogen transformations (e.g., Chin-Leo and Benner, 1992; Pakulski et al., 1995; Pomeroy et al., 1995; Jochem, 2001, 2003; Liu et al., 2004, 2009; Malmstrom et al., 2004; Hewson et al., 2006), and shown that bacteria form critical linkages within the food webs of these systems (Dagg et al., 2006). Carbon and nitrogen transformations are particularly important, not only because of their contributions to the “microbial loop,” but because they affect the development and persistence of an extensive oxygen-depleted or hypoxic zone (Pakulski et al., 1995; Chen et al., 2001; Dagg et al., 2006). This zone, which develops seasonally and threatens the integrity of both planktonic and benthic systems on the nGoM shelf, depends on elevated primary production supported by riverine nitrogen inputs, and high bacterial respiration rates that reduce oxygen concentrations.

Additional studies have also shown that nGoM bacterioplankton mediate the impacts of acute and chronic anthropogenic disturbances to nGoM waters, including nitrogen loading and aromatic and aliphatic hydrocarbon inputs from a variety of sources (Hall et al., 2008; Hazen et al., 2010; Senn et al., 2010; Valentine et al., 2010; Edwards et al., 2011; Kessler et al., 2011). Recent data derived from assessments of the Deep-water Horizon (DWH) oil spill have shown that bacterioplankton within an oil and gas plume at depths of about 1000–1200 m responded rapidly to hydrocarbon inputs, were dominated by γ-Proteobacteria, included taxa most closely related to genera known for hydrocarbon degradation, and were distinct from non-plume bacterioplankton.

Nonetheless, surprisingly little is known about nGoM bacterioplankton composition and structure. Jones et al. (2010) have shown that Proteobacteria dominate bacterioplankton in near-shore waters of the west Florida shelf, and that community compositions change during blooms of *Karenia brevis*. Olapade (2010) also identified α-Proteobacteria as dominant members of nGoM bacterioplankton, but his sites were all in shallow near-shore waters, and only one (from Carrabelle) was characterized by seawater salinities. However, this site cannot be used to extrapolate to the nGoM broadly since it is adjacent to a developed beach (Olapade, 2010).

In an effort to characterize nGoM bacterioplankton communities, we have analyzed 44 samples obtained during mid-March 2010 at 17 stations distributed along a longitudinal gradient with transects from inshore to offshore, including the Mississippi River plume, and depths from 2 to 1700 m. Results showed that α- and γ-Proteobacteria, Bacteriodetes, and Actinobacteria dominated surface (≤100 m) communities, with minor contributions from Planctomycetes and Verrucomicrobia. α- and γ-Proteobacteria also dominated deeper communities (>100 m), but these communities included relatively large fractions of Thaumarchaeota. Although members of the SAR11 clade were abundant in all but a Mississippi River plume surface sample, the distribution of OTUs identified at an evolutionary distance of 0.03 varied substantially among sites, with relatively few taxa shared widely.

## Materials and Methods

### Sample collection and DNA extraction

**Figure A1:**
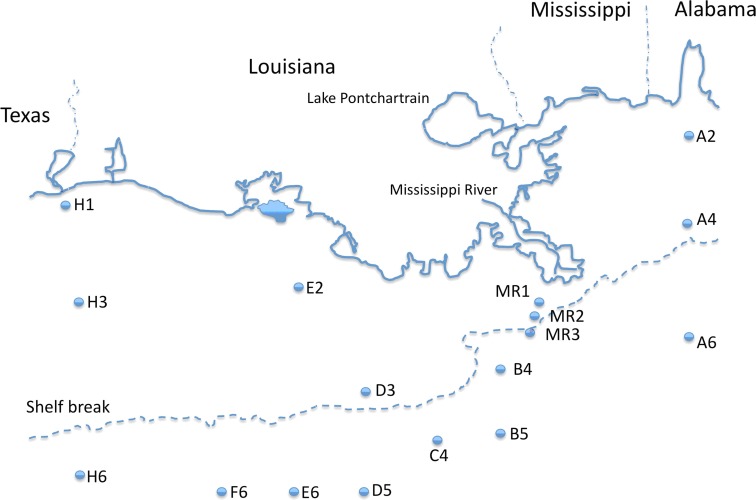
**Station locations for samples used in this study**.

Samples were collected during March 2010 during *R/V Cape Hatteras* cruise GC-5 as described by Tolar et al., in revision; see also Figure A1 in Appendix for station locations and Table A1 in Appendix for selected sample data). Samples were obtained using Niskin bottles and a General Oceanics rosette sampling system equipped with a SBE25 CTD data package. They were pressure filtered through 0.22 μm Durapore filters (about 1 l at ∼60 kPa; Millipore, Inc., New Bedford, MA, USA). Filters were frozen at −20°C in 2 mL lysis buffer (0.75 M sucrose, 40 mM EDTA, 50 mM Tris; pH 8.3). After enzymatic hydrolysis (lysozyme and proteinase K with sodium dodecyl sulfate), DNA in 800 μL of lysate was purified by phenol-chloroform extraction. Purified DNA was stored in 50 μL of Tris-EDTA buffer (pH 8) at −80°C (Bano and Hollibaugh, 2002).

### PCR and pyrosequencing

Triplicate PCR reactions for each sample consisted of 12 μL of PCR grade water, 2.5 μL 10× High Fidelity PCR Buffer (Invitrogen), 0.75 μL 25 mM dNTP mix, 1 μL 50 mM MgSO_4_, 5 μL 5× Bovine Serum Albumin (Promega), 1.5 μL each of forward and reverse primers (10 mM stocks), 0.2 μL Platinum Taq DNA Polymerase High Fidelity (Invitrogen), and 0.5 μL template DNA. Primers 515F and 806R were used to amplify Bacteria and Archaea 16S rRNA genes (Liu et al., 2007; Jones et al., 2009; Bergmann et al., 2011). Primer 515F included the Roche 454-B pyrosequencing adapter and a GT linker. Primer 806R included the Roche 454-A sequencing adapter, a 12-bp unique barcode (Hamady et al., 2010), and a GG linker. The PCR program consisted of an initial denaturation step (94°C; 3 min), followed by 26 cycles of 94°C (1 min), 54°C (1 min), 68°C (2 min), with a final extension of 10 min at 68°C. Amplicons were visualized by electrophoresis on a 1% agarose gel. Products from triplicate reactions were pooled and purified with UltraClean PCR Clean-up kits (MoBio; Folsom, CA, USA) according to the manufacturer’s instructions. After quantifying DNA concentrations of the cleaned reactions using a Nanodrop spectrophotometer, equal masses of PCR product for each sample were combined and shipped to the Environmental Genomics Core Facility at the University of South Carolina where pyrosequencing was performed using a Roche 454 automated sequencer using titanium chemistry.

### Data analysis

Sequences were analyzed using PANGEA (Giongo et al., 2010) and Mothur (Schloss et al., 2009) pipelines. After trimming the initial set of reads (435,290 sequences), PANGEA produced 239,983 sequences (>150 bp and ≥20 quality score) with identifiable barcodes distributed among the 44 samples (minimum sequence number per sample = 764, maximum = 9154; mean = 5454, SE = 362). A total of 38,045 reads could not be associated with a barcode. PANGEA identified the phylogenetic affiliations of the reads using MEGABLAST with a database of 170,273 Bacteria and Archaea isolate 16S rRNA sequences obtained from the Ribosomal Database Project. PANGEA clustered unidentified sequences for each sample using CD-HIT and threshold values (*D*) of 0.80, 0.90, 0.95, 0.97, and 0.99. OTUs comprised of sequences that could be identified with MEGABLAST were clustered similarly for each sample, and these clusters were combined with unidentified OTUs to produce the final composition for each sample. Spatial patterns in the resulting sample compositions, including OTU abundances, were analyzed using multivariate statistics [e.g., principal components analysis (PCA), non-metric multidimensional scaling, and canonical correlation analysis (CCorA)] with XLSTAT after excluding cyanobacterial and mitochondrial sequences, and removing OTUs represented by singletons within the 44-sample set.

Sequences were also processed using the Mothur platform (Schloss et al., 2009). After trimming barcodes and primers, and filtering for quality, sequences ≥150 bp were aligned to the Greengenes ProkMSA aligned database. Chimeric sequences were identified with the Chimera Slayer algorithm from the Broad Institute and removed. The pre.cluster command was used to minimize errors introduced during pyrosequencing. Cyanobacterial sequences were excluded from analysis. A total of 109,867 sequences comprised of 15,071 unique sequences were included in the analysis. For OTU-based analyses of sample composition, the “average neighbor” clustering algorithm was used to group sequences at a similarity level of 97%. The taxonomy function in Mothur was used with the Ribsosomal Database Project (RDP) training set to identify each OTU for phylotype-based. Because the RDP database used for analysis lacked Thaumarchaeota sequences, and because this phylum is highly represented in the Gulf of Mexico (Tolar et al., in revision), representative sequences for all OTUs that were identified as Archaea at a distance of 0.03 were analyzed with BLAST against the Greengenes prokMSA database to verify their identity. We also used the NCBI database to conduct a manual BLAST analysis of the representative sequences for the most abundant OTUs at a distance of 0.03 to verify identities, and to assess membership within SAR11, SAR86 and SAR92, and SAR324 clades for α-, γ-, and δ-Proteobacteria, respectively. These results were further verified by analyzing representative sequences in the ARB-SILVA database using the classify function. Mothur was also used to generate diversity indices based on samples with normalized numbers of sequences, to select specific phylogenetic groups for further analysis and to provide input for fast UniFrac (Hamady et al., 2010), which was used to assess spatial patterns in bacterioplankton communities.

The phylogenetic structure of nGoM bacterioplankton was also analyzed using Phylocom (Webb et al., 2002) to calculate the “net relatedness index” (NRI) and “nearest taxon index” (NTI). NRI is a standardized estimate of the mean pairwise phylogenetic distances for all pairs of OTUs in a sample compared to the mean pairwise distances of a random or null set of OTUs; NRI provides a measure of the extent of tree-wide clustering, including terminal and deep branches. NTI is a similarly standardized measure, but assesses the phylogenetic distance of each OTU in a sample to its nearest neighbor OTU; NTI provides a measure of terminal clustering independent of deeper clustering (Webb et al., 2002). Both indices were generated with 9999 randomized runs and included OTU frequencies.

Sequence data have been deposited with MG-RAST (metagenomics.anl.gov) at accession numbers 4509220.3–4509263.3. Metadata are available via the project page, “Analysis of composition and structure of coastal to mesopelagic bacterioplankton communities in the nGoM.”

## Results

**Figure 1:**
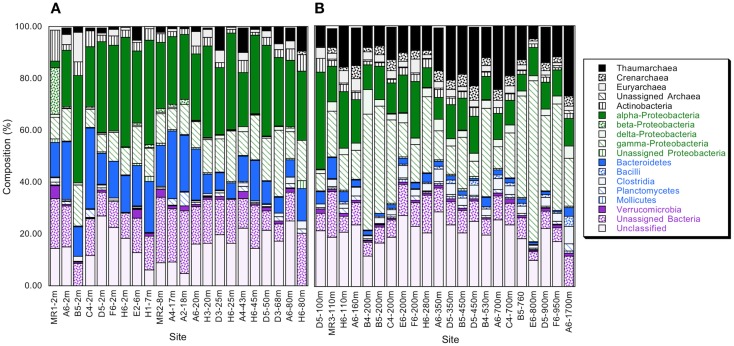
**(A)** Relative abundance of phyla and classes for bacterioplankton samples obtained from depths ≤100 m as determined from PANGEA analysis, excluding chloroplast, and cyanobacterial sequences; minor phyla and classes represented by sequences accounting for ≤0.1% of the total for each sample are not shown. **(B)** As for **(A)**, but data are for samples from >100 m. Station positions are mapped in Figure A1 in Appendix.

**Figure 2:**
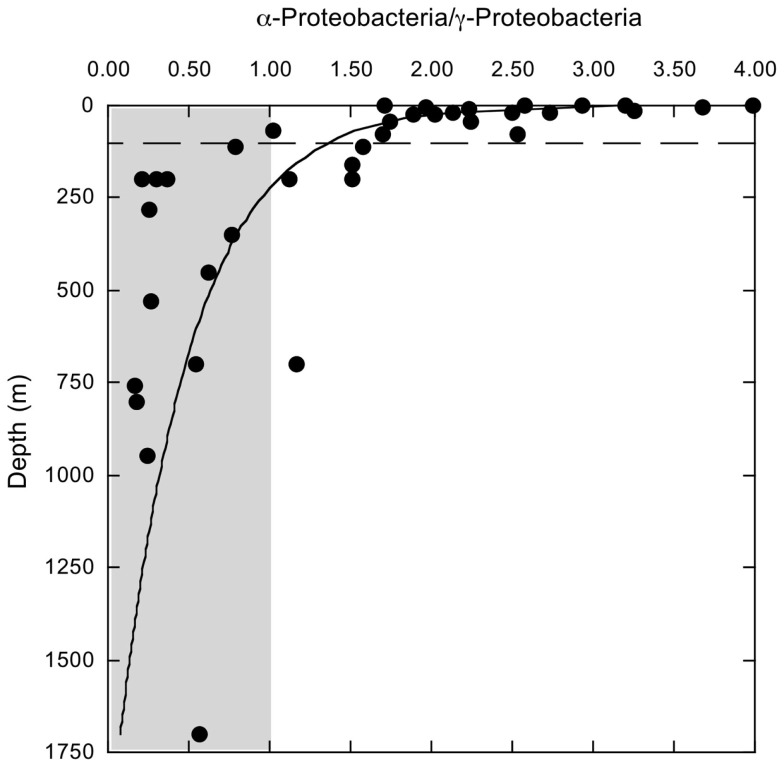
**Variation with depth in the ratios of α-proteobacteria/γ-Proteobacteria sequences identified by PANGEA for each of 44 samples**. The dashed line indicates a depth of 100 m. Shaded area indicates values <1.

Bacterioplankton community composition varied substantially with depth, but relatively little among stations. The most divergent station, MR1–2 m, was represented by a low salinity surface sample (2 m depth, 0.54 ppt) of the Mississippi River plume; it was comprised of very low and high percentages of α- and β-Proteobacteria (2.5 and 18.8%), respectively (Figure 1A). MR1-2 m also supported relatively high percentages of Verrucomicrobia (5.0%). Bacterioplankton compositions for the remaining samples from depths ≤100 m (Figure 1A) were characterized by α- and γ-Proteobacteria, Bacteroidetes, and Thaumarchaeota with relative abundances of 30.27 ± 1.41, 12.58 ± 0.97, 14.00 ± 1.51, and 3.10 ± 0.69% (mean ± 1 SE), respectively. These same groups occurred at 12.06 ± 0.88, 24.39 ± 3.23, 2.26 ± 0.36, and 14.11 ± 1.33%, respectively, for deeper communities (Figure 1B). The differences in relative abundances for each group above and below 100 m were highly significant (*t*-test, *p* < 0.001). The trends in Proteobacteria resulted in a distinct change in the ratios of α-Proteobacteria/γ-Proteobacteria (α/γ) above and below 100 m (Figure 2). At depths ≤100 m α/γ ratios were >1.7 (mean = 2.69, SE = 0.24) with a single exception; ratios at depths >100 m were all < 1.6 (mean = 0.77, SE = 0.13).

Although the relative abundances of major phyla and sub-phyla (or classes) changed with depth, members of the ubiquitous SAR11 clade dominated the α-Proteobacteria throughout the water column, accounting for about 69% of all α-Proteobacteria sequences. Representatives of *Alteromonas* and *Pseudoalteromonas* dominated the γ-Proteobacteria, with contributions of 31 and 6%, respectively, by members of the widely distributed SAR86 and SAR92 clades. These groups did not vary consistently with depth or geographic location.

**Figure 3:**
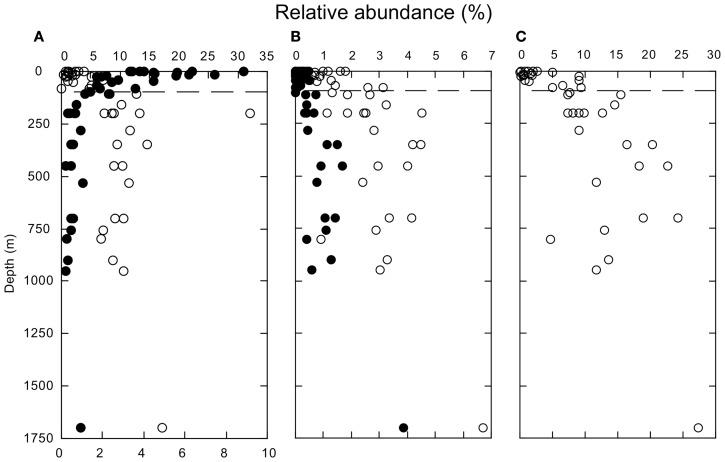
**(A)** Depth profiles of the relative abundances of Bacteroidetes (closed symbols) and δ-Proteobacteria (open symbols). **(B)** Depth profiles of the relative abundances of Bacilli (closed symbols) and Clostridia (open symbols). **(C)** Depth profiles of the relative abundances of Thaumarchaeota. All identifications based on PANGEA analysis. The dashed line indicates a depth of 100 m.

**Figure A2:**
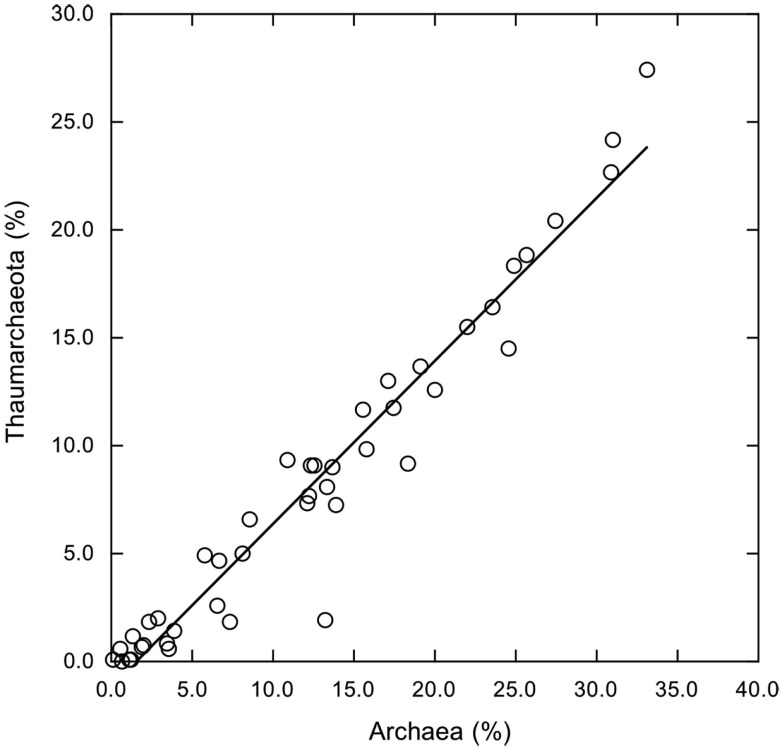
**Thaumarchaeota sequences as a percentage of total sequences plotted versus total Archaea sequence percentages**. Linear regression trend line indicates that Thaumarchaeota account for about 76% of all Archaea sequences (*y* = −1.14 + 0.755×, *r*^2^ = 0.946) regardless of sample site or depth.

Several other groups exhibited consistent variations in relative abundance with depth. Relative abundances of δ-Proteobacteria, Bacilli, and Clostridia were low in surface waters, but increased below 100 m (Figures 3A,B). In spite of their anaerobic character, δ-Proteobacteria, and Clostridia represented ∼2–4 and 1–6%, respectively, of the sequences from oxic deep-water samples. Most of the sequences affliliated with δ-Proteobacteria (about 66%) belonged to the SAR324 clade. In addition, Thaumarchaeota increased in relative abundance below 100 m (Figure 3C). Thaumarchaeota accounted for ∼10–25% of deep-water sequences in general, and 27% of sequences at a 1700-m deep site; they also constituted a relatively constant fraction of the Archaea (about 75%, Figure A2 in Appendix), with unclassified Archaea and a small percentage of Euryarchaeota accounting for the remainder.

**Figure A3:**
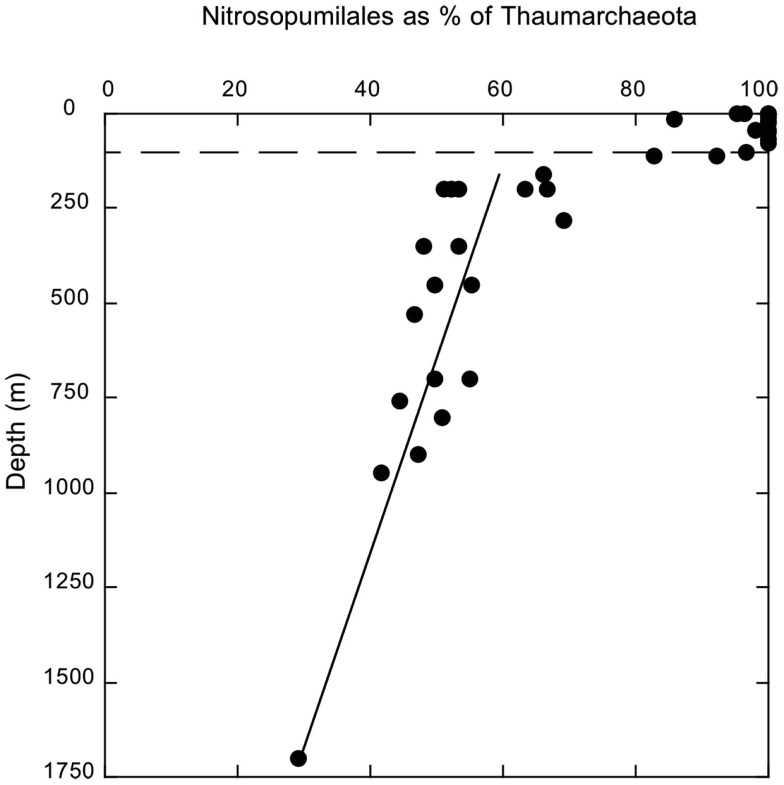
**Depth distribution of Nitrosopumilales sequences as a percentage of Thaumarchaeota sequences**. Dashed line indicates 100 m depth.

Thaumarchaeotal sequences were comprised of two clades: one contained sequences that were most closely related to a shallow water/sediment group of Nitrosopumilales, while the second consisted of sequences most closely related to a deep-water group of Cenarchaeales. The former accounted for most (80–100%) of the Thaumarchaeota sequences in surface waters, but the contribution of this clade decreased linearly (*r*^2^ = 0.634, *p* < 0.0001) with increasing depth below 110 m, reaching a minimum of 29% at 1700 m (Figure A3 in Appendix).

**Figure 4:**
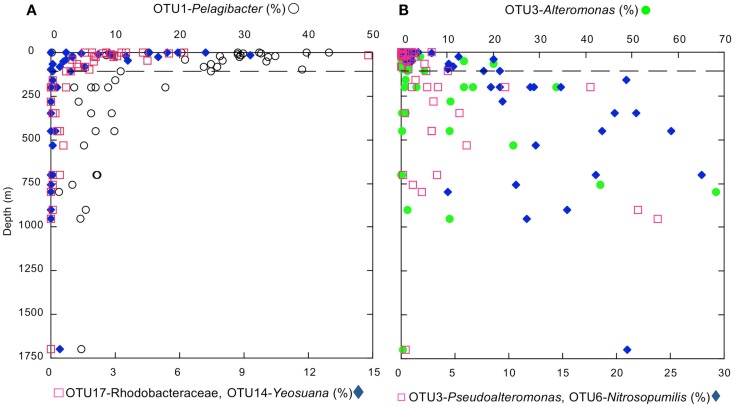
**(A)** Depth profiles of the most abundant individual α-proteobacteria (*Candidatus Pelagibacter ubique* and a Rhodobacteraceae) and Bacteroidetes (*Yeosuana* sp.) OTUs identified by PANGEA. **(B)** Depth profiles of the most abundant individual γ-Proteobacteria (*Alteromonas* and *Pseudoalteromonas*) and Thaumarchaeota (*Nitrosopumilis* sp.) OTUs identified by PANGEA. The dashed line indicates a depth of 100 m.

The distributions of the 10 most abundant OTUs (defined at an evolutionary distance = 0.03), which collectively accounted for >44% of the sequences analyzed by the Mothur pipeline, were consistent with distributions at phylum and class levels (e.g., Figure 4A). An OTU identified as *Candidatus Pelagibacter* *ubique* (SAR11 clade, α-Proteobacteria) occurred in all samples, and dominated the bacterioplankton at depths ≤110 m; however at greater depths, its relative abundance declined markedly. Similar profiles were observed for other α-Proteobacteria OTUs (e.g., Rhodobacteriaceae), and for an OTU identified as *Yeosuana* (Bacteroidetes). In contrast, OTUs identified as γ-Proteobacteria (e.g., *Alteromonas*, *Pseudoalteromonas*, and a representative of SAR86) and Thaumarchaeota increased in relative abundance below 100 m, although their distribution was variable (Figure 4B).

**Figure 5:**
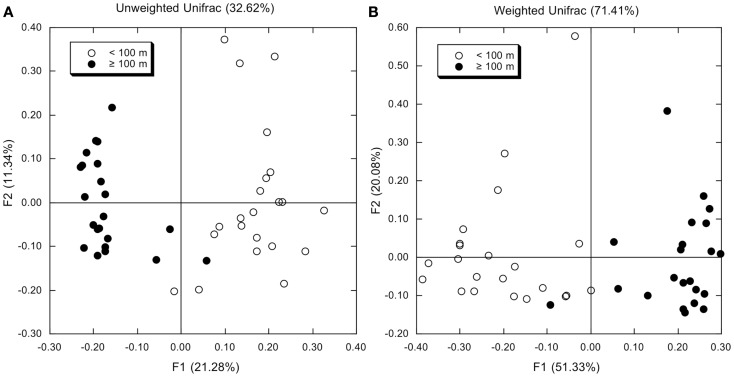
**(A)** Results from a principal components analysis of unweighted UniFrac distances determined using OTUs identified by Mothur analysis (distance = 0.03) for each bacterioplankton sample. Open symbols represent samples from depths <100 m; closed symbols represent samples from depths ≥100 m. **(B)** As for **(A)**, but using weighted UniFrac distances.

**Figure 6:**
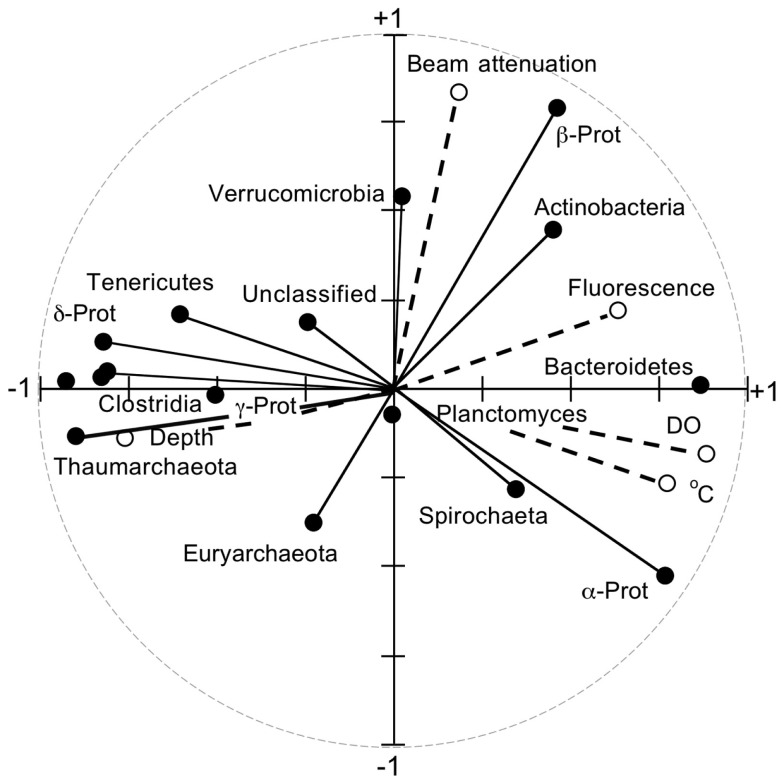
**Results from a canonical correlation analysis using relative abundances of phyla and classes (e.g., as in Figure 1) as determined by PANGEA, and salinity, pH, depth, dissolved oxygen, fluorescence (a measure of chlorophyll concentration), and beam attenuation (a measure of particle content) for each sample**.

**Figure A4:**
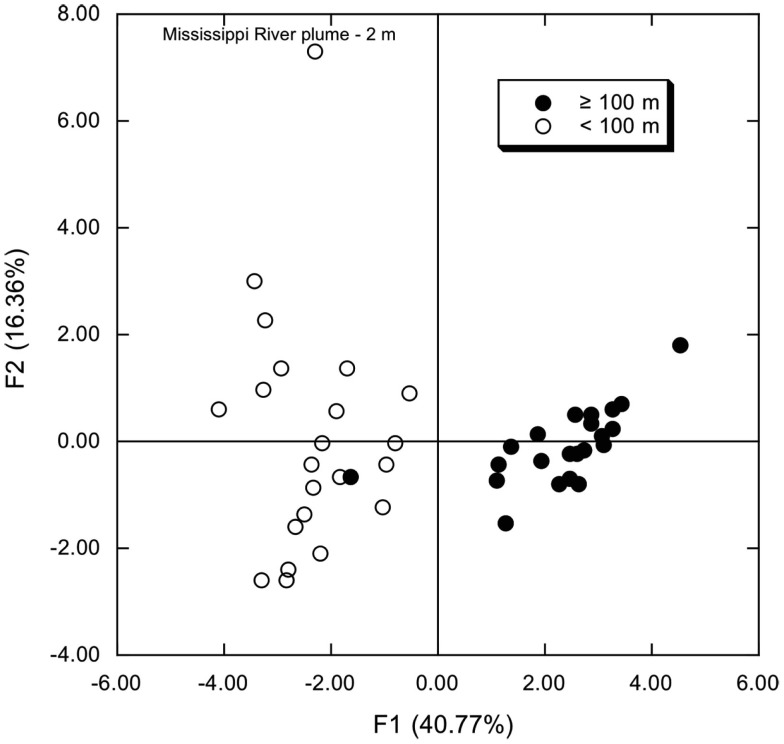
**Principal components analysis of phylum and class compositions for all stations and depths; percentages were analyzed after an arcsine transformation**. Open symbols represent samples from depths <100 m; closed symbols represent samples from depths ≥100 m.

Principal component analyses based on UniFrac indices (Figure 5) and composition (Figure A4 in Appendix) revealed two sample clusters distinguished on the first PCA axis. These two clusters were comprised of samples from depths ≤100 and >100 m, respectively, and were consistent with trends observed in depth profiles of bacterioplankton composition (e.g., Figures 1, 3, and 4). Within each of these clusters samples were not further differentiated based on depth or location, with the exception of station MR1–2 m, which consistently differed from all other samples. A canonical correlation analysis indicated that Clostridia, δ- and γ-Proteobacteria, and Thaumarchaeota were positively correlated with depth, and α-Proteobacteria were inversely correlated with depth (Figure 6). α-Proteobacteria and Bacteroidetes were positively correlated with temperature and dissolved oxygen, while Actinobacteria, β-Proteobacteria, and Verrucomicrobia were positively correlated with beam attenuation, a measure of suspended particles in the water column.

**Figure 7:**
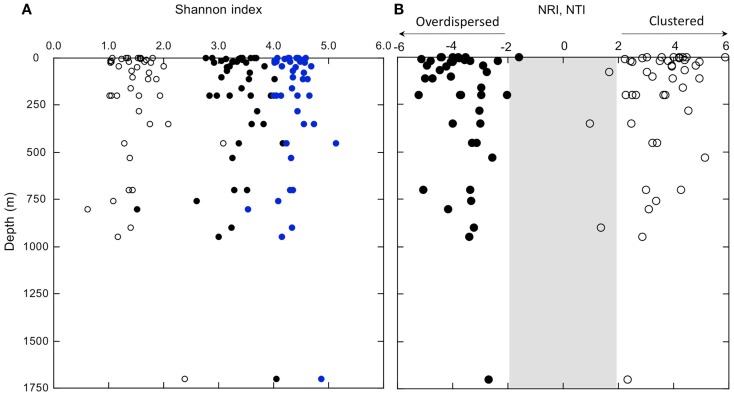
**(A)** Shannon indices determined using Mothur analysis of OTUs (distance = 0.03) in normalized samples (equal read numbers); open circles represent theoretical minimum estimates, closed symbols represent observed values, and open diamonds represent theoretical maximum values (see text). **(B)** Depth profiles of NRI (closed symbols) and NTI (open symbols); values within the shaded region are not statistically significant.

**Figure A5:**
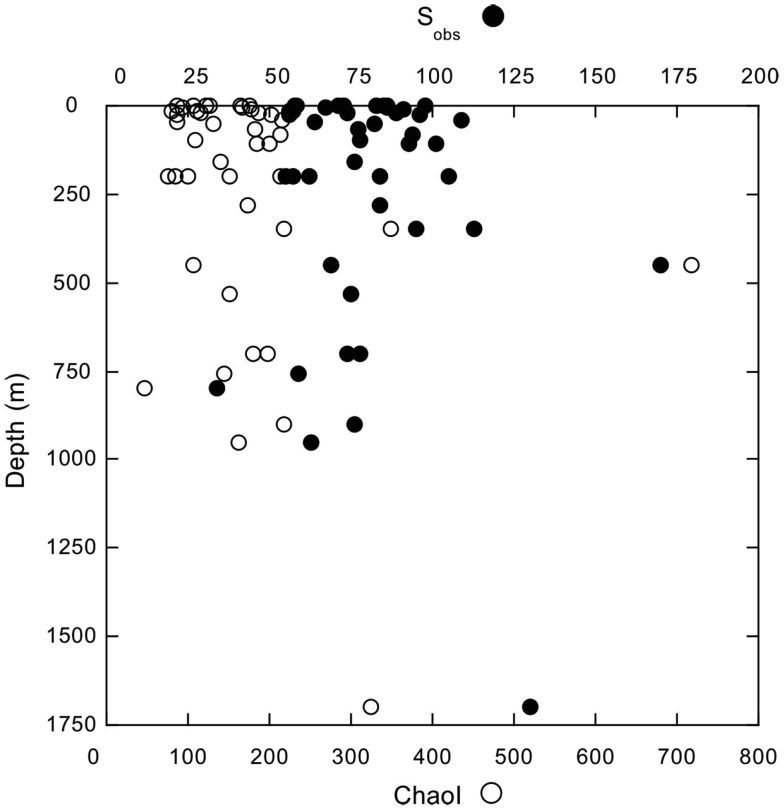
**Depth distributions of observed OTU richness (*S*_obs_, *d* = 0.03) and the Chao1 index derived from Mothur**.

In spite of consistent differences in bacterioplankton composition between surface and deeper waters, diversity indices were not spatially structured. Neither richness indices (Figure A5 in Appendix) nor evenness and dominance indices (Shannon and inverse Simpson’s) correlated significantly with depth (Figure 7A; Table A2 and Figure A5 in Appendix). Likewise, two measures of phylogenetic community structure, the NRI and NTI, did not vary consistently with depth (Figure 7B). NRI values were ≤−1.96 for most samples, which indicated that communities were significantly overdispersed. Overdispersion occurred because the OTUs of any given community were less related to each other in a community-wide phylogenetic comparison than expected for a community assembled randomly from a given pool of OTUs. In contrast, NTI values were mostly >1.96. This indicated that communities were significantly clustered. Clustering occurred because in any given community the *nearest* phylogenetic neighbors of each of its OTUs were more closely related than expected for a randomly assembled community (Figure 7B).

## Discussion

### Depth-related changes in composition

This first detailed assessment of nGoM bacterioplankton spatial variability, diversity, and phylogenetic community structure reveals that composition varies substantially between surface (≤100 m) and sub-surface (>100 m) samples (e.g., Figures 1A,B and 6). Although changes in community composition with depth typify marine systems (e.g., Lin et al., 2006; De Corte et al., 2009; Church et al., 2010; Agogue et al., 2011; Eiler et al., 2011; Zinger et al., 2011; Friedline et al., 2012), the observed distribution of nGoM Proteobacteria differs from patterns reported by others. For example, in most other systems, α-Proteobacteria abundance typically exceeds that of γ-Proteobacteria regardless of depth (e.g., Cottrell and Kirchman, 2000; Pommier et al., 2007; Barberán and Casamayor, 2010; Galand et al., 2010; Zinger et al., 2011). While this trend holds for nGoM surface samples (≤100 m), γ-Proteobacteria abundance exceeds that for α-Proteobacteria in most nGoM deep-water sites, in some cases by >2-fold (Figure 2). Similar relationships have also been described for bathypelagic bacterioplankton in the Sea of Marmara (Quaiser et al., 2011) and the deep Arctic Ocean (Galand et al., 2010), but factors that account for this similarity are unclear.

The controls of nGoM α-Proteobacteria distribution are uncertain, as are reasons for the increased relative abundance of γ-Proteobacteria with depth. Multiple linear regression analyses show that neither depth, temperature, dissolved oxygen, pH, fluorescence (a measure of chlorophyll concentration), nor particle concentration (beam attenuation) individually accounts for α-Proteobacteria variability. However, the interaction of temperature and oxygen explains >70% (*r*^2^ = 0.760, *p* < 0.0001). While this suggests that α-Proteobacteria might be sensitive to the combined effects of several variables that affect metabolic activity, factors other than or in addition to those included here likely play important roles.

Both the individual variables noted above and interactions among variables have limited explanatory power for γ-Proteobacteria distributions (e.g., maximum *r*^2^ = 0.244, *p* = 0.003). This indicates that nGoM α- and γ-Proteobacteria respond to different ecological determinants. The availability of aliphatic and aromatic hydrocarbons emanating from extensive hydrocarbon deposits on the nGoM continental slope (Sassen et al., 2001; Milkov and Sassen, 2003; Liu et al., 2009) might account for the increased relative abundance of γ-Proteobacteria in deeper waters of the nGoM compared to other systems. Several prior studies in other systems have shown that γ-Proteobacteria respond strongly to hydrocarbon availability (Gerdes et al., 2005; Al-Awadhi et al., 2007; Berthe-Corti and Nachtkamp, 2010), and Hazen et al. (2010), Valentine et al. (2010), Kessler et al. (2011) have shown that various γ-Proteobacteria formed blooms at depths of about 1000–1200 m in response to the DWH hydrocarbon plume. In support of a role for hydrocarbon availability as a structuring factor for deep γ-Proteobacteria, members of the hydrocarbon-oxidizing genus, *Pseudoalteromonas* (Melcher et al., 2002), are among the most abundant phylotopes in deep nGoM waters (Figure 4B).

The increase in relative abundance of both γ- and δ-Proteobacteria with depth (Figures 3A and 4B) might also be supported by chemolithoautotrophic growth as proposed by Swan et al. (2011). However, Swan et al. (2011) characterized clades of γ-Proteobacteria (ARCTIC96BD-19 and Agg47) that are poorly represented in the nGoM, and it is not yet known if some of the better represented groups, e.g., SAR86 and SAR92, grow chemolithoautotrophically. Initial genomic analyses suggest that SAR86 lacks genes essential for CO_2_ fixation (Dupont et al., 2012). In contrast, Swan et al. (2011) provided evidence for chemolithoautotrophic metabolism in a δ-Proteobacteria clade that is important in deep-water nGoM communities. This clade, SAR324, accounts for about 66% of nGoM δ-Proteobacteria. Thus, chemoautolithotrophy might account for changes in δ-Proteobacteria, while other factors determine the distribution of γ-Proteobacteria.

The abrupt increase in Thaumarchaeota relative abundance below 100 m as reported (Figure 3C) here agrees with results of a separate qPCR study of Archaea and Bacteria 16S rRNA genes at the same sites (Tolar et al., in revision). Tolar et al. (in revision) show that compared to Bacteria, absolute and relative Thaumarchaeota abundances increase in deep samples. An earlier study of nGoM Archaea at a single station (samples from 10, 400, 900 m) also observed that Marine Group I Crenarchaeota (i.e., Thaumarchaeota) dominate at depth, while Group II-β Euryarchaeota dominate in surface waters (Liu et al., 2009).

Although multiple reports have documented increased Thaumarchaeota abundance with increasing water column depth, the controls of this pattern remain unclear. Some have proposed that Thaumarchaeota are better adapted to environments characterized by low metabolic energy fluxes (see Valentine, 2007), and thus are better able than other lineages to couple growth to the limited resources (e.g., ammonium and perhaps some heterotrophic substrates) available below the epipelagic zone (Pester et al., 2011).

This hypothesis has implications for the spatial partitioning of thaumarchaeal clades reported by others (Santoro et al., 2010; Hu et al., 2011a,b; Yakimov et al., 2011) and also observed here (Figure A3 in Appendix). In particular, a “shallow water” clade consisting of phylotypes from surface waters and sediments has been distinguished from a “deep-water” clade found largely in meso- to bathypelagic samples (Beman et al., 2008; Hu et al., 2011a,b; Tolar et al., in revision). For nGoM samples, deep-clade phylotypes increase in relative abundance abruptly below 100 m, and then continue to increase linearly with depth (Figure A3 in Appendix). This might indicate that the surface-clade phylotypes are less well adapted than deep-clade phylotypes to low metabolic energy fluxes. Differences in gene expression for members of each clade in surface and deep samples could provide an indication of adaptations and competitiveness.

### Spatial stability of diversity with depth

Although nGoM bacterioplankton composition changes substantially with depth, neither richness (e.g., Chao 1), nor evenness (e.g., Shannon index) and dominance (e.g., inverse Simpson’s index) measures of diversity show any distinct geographic trend (Figure 7A; Table A2 and Figure A5 in Appendix). The absence of trends within nGoM bacterioplankton at a regional scale is consistent with patterns reported for samples at a global scale. In particular, no distinct general trends as a function of depth or location have been reported for Chao 1 and the Shannon and inverse Simpson’s indices for assemblages ranging from epipelagic to bathypelagic depths in the sub-tropical Pacific to the Arctic Ocean (summarized in Table 2, Stevens and Ulloa, 2008), although site-specific trends have been described for Archaea and Bacteria (e.g., Brown et al., 2009). This similarity suggests common patterns for community assembly regardless of location, depth, temperature, or other variables. Comparisons among data sets should be treated cautiously, however, since diversity indices have well known limitations (Hill et al., 2003). These include sensitivity to sequencing effort, numbers of OTUs, and patterns of rank abundance within a given community. Nonetheless, Hill et al. (2003) have shown that Chao 1 and the Shannon index can provide robust measures of changes among samples or systems.

To promote comparisons for the nGoM, we have used the Mothur pipeline (Schloss et al., 2009) to produce normalized samples comprised of equal numbers of sequences randomly selected from the pool of sequences available for each sample. We have then compared theoretical minimum and maximum Shannon indices with observed values (Figure 7A). Minimum values assume a single dominant OTU, and that all others occur as singletons. Maximum values assume that all OTUs are equally abundant. With one notable exception, a sample from 760 m, most of the assemblages are characterized by Shannon indices that range between about 50 and 70% of theoretical maxima. This provides further support for the notion that the structure of nGoM bacterioplankton communities develops independently of major physical-chemical variables (e.g., depth, temperature, oxygen) and even some biological variables (e.g., community composition, chlorophyll concentration).

### Spatial stability of phylogenetic community structure

Two measures of phylogenetic community structure, NRI and NTI, provide additional insights about nGoM bacterioplankton communities. For this study, NRI values have been calculated using the full set of nGoM OTUs for null models; however, the choice of null model (see http:/phylodiversity.net/phylocom/phylocom_manual.pdf) did not affect the outcome.

Like nGoM diversity indices, neither NRI nor NTI vary consistently spatially (Figure 7B). This suggests that substantial changes in numerous physical-chemical and biological variables have little effect on phylogenetic community structure. Negative NRI values for all samples indicate that nGoM bacterioplankton communities are phylogenetically overdispersed throughout the water column. Overdispersion arises in a community when its OTUs are more distantly related to each other across terminal and deep branches of a community phylogenetic tree than expected by chance. Multiple processes have been proposed to account for overdispersion, including competitive exclusion when traits that determine community membership are phylogenetically conserved, and habitat filtering when such traits arise in different lineages by convergence or horizontal gene transfer (Webb et al., 2002; Vamosi et al., 2009).

Distinguishing between these or other processes that might lead to overdispersion requires a deeper understanding of the factors that constrain bacterioplankton richness than is currently available. However, since oligotrophs dominate the marine microbiota (Lauro et al., 2009), heterotrophic substrate limitation might promote competitive exclusion, particularly if different groups of bacteria specialize on different carbon sources. Lauro et al. (2009) have recently identified genomic/metagenomic markers for copiotrophs and oligotrophs. Phylogenetic analysis of these markers in nGoM and other bacterioplankton could provide a means to test relationships between substrate utilization and phylogenetic community structure.

In contrast to uniformly negative NRI values, uniformly positive NTI values (Figure 7B) indicate that nGoM OTUs occurring at the tips of the community trees form clusters more closely related than expected by chance. OTU clustering has been attributed to “habitat filtering,” which occurs when one or more environmental variables determine the patterns of community assembly. However, since clustering characterizes all nGoM samples, the environmental variables responsible for habitat filtering are uncertain, and likely vary among phylogenetic groups and with depth.

Habitat filtering has also been suggested as a structuring agent for surface bacterioplankton communities based on analyses of 16S rRNA gene sequences obtained from globally distributed samples (Barberán and Casamayor, 2010; Pontarp et al., 2012). For many of these communities, positive values have been obtained for both NRI and NTI, although in some cases similar to the nGoM, negative NRI, and positive NTI values have also been reported. The more consistently positive NRI values in global scale studies (Barberán and Casamayor, 2010; Pontarp et al., 2012) might have arisen in part from differences in the null communities used to calculate NRI (Webb et al., 2002). Null communities used in global scale studies have been derived from a global OTU pool, while the nGoM analysis has been based on a regional pool. Nonetheless, NTI values from all samples are consistent with environmental selection as a driver for assembly of both surface and deep communities.

## Summary and Conclusion

An extensive analysis of nGoM bacterioplankton (17 stations, 44 discrete samples, depths from 2 to 1700 m) showed that distinct assemblages characterized ≤100 and >100 m depths. SAR 11 α-Proteobacteria and Bacteroidetes were prominent in the former, while γ-Proteobacteria, Firmicutes, and Thaumarchaeota were prominent in the latter. Though composition varied substantially with depth, diversity indices did not, which indicated that the structure of nGoM bacterioplankton communities was relatively stable across large gradients in physical-chemical and biological variables. Phylogenetic community structure was also relatively stable, with no variation evident among stations or depths. NTI values indicated that habitat filtering played a role in community assembly at all depths, while NRI values indicated that other processes, e.g., competitive exclusion, contributed as well. Collectively, these results offer the first synoptic insights into the composition and diversity of nGoM bacterioplankton, and provide a basis for understanding their dynamics at a regional scale.

## Conflict of Interest Statement

The authors declare that the research was conducted in the absence of any commercial or financial relationships that could be construed as a potential conflict of interest.

## Appendix

**Table A1:** **Selected physical-chemical variables for samples used in this study; DO, dissolved oxygen; Beam atten, beam attenuation**.

Station	Depth (m)	Temp (°C)	Salinity ppt	DO (mg l^−1^)	Fluoresence	Beam atten	pH
A2	18	16.3	33.345	7.76	3.32	0.95	8.16

A4	17	17.2	34.981	7.72	1.65	0.60	8.17
	43	19.0	36.278	5.84	0.90	1.16	8.10

A6	2	19.1	35.899	6.15	1.32	0.42	7.74
	20	19.0	35.997	7.19	1.34	0.43	7.74
	80	19.4	36.416	6.61	0.37	0.44	7.76
	160	17.0	36.248	4.08	0.14	0.46	7.83
	350	9.8	35.175	3.66	0.13	0.50	8.14
	700	6.4	34.902	4.42	0.09	0.57	8.16
	1700	4.3	34.968	6.64	0.07	0.58	8.17

B4	200	13.6	35.721	3.79	0.12	0.50	NA
	530	7.2	34.926	3.98	0.16	0.53	7.72

B5	2	19.5	36.454	6.91	1.19	0.53	8.13
	200	16.7	36.218	4.27	0.11	0.46	7.95
	450	9.9	35.182	3.55	0.11	0.46	7.75
	760	6.0	34.906	4.68	0.14	0.50	7.74

C4	2	18.4	34.462	7.82	4.89	1.09	8.20
	200	15.4	36.007	4.14	0.11	0.46	7.98
	700	6.9	34.915	4.17	0.10	0.49	7.73

D3	25	18.5	35.897	6.47	1.11	0.62	8.11
	68	18.6	36.437	6.55	0.49	4.01	8.11

D5	2	19.7	36.482	6.94	1.03	0.55	8.14
	50	19.7	36.481	6.92	1.19	0.55	8.14
	100	19.5	36.472	6.18	0.27	0.46	8.11
	350	10.7	35.292	3.58	0.12	0.44	7.78
	450	8.7	35.037	3.70	0.10	0.44	7.74
	900	5.6	34.914	5.05	0.09	0.45	7.73

E2	6	16.4	32.096	4.75	2.97	4.33	8.20

E6	200	14.9	35.924	4.13	0.11	0.45	7.90
	800	5.9	34.908	4.77	0.09	0.46	7.72

F6	2	20.3	36.434	7.08	1.22	0.60	8.16
	200	15.9	36.086	4.06	0.16	0.49	7.93
	950	5.2	34.926	5.36	0.09	0.45	7.72

H1	7	15.1	26.976	8.43	9.55	5.20	8.24

H3	20	16.6	35.065	7.03	1.57	1.28	8.13

H6	2	20.0	36.429	7.13	0.63	0.58	8.16
	25	19.5	36.390	7.25	1.20	0.61	8.15
	45	19.2	36.380	6.97	1.42	0.56	8.11
	80	18.3	36.388	6.07	0.51	0.51	8.08
	110	17.5	36.321	5.20	0.18	0.51	8.00
	280	12.4	35.543	3.52	0.13	0.50	7.82

MR1	2	10.6	0.543	6.91	3.35	21.70	7.83

MR2	8	17.8	30.355	6.80	2.56	3.26	7.88

MR3	110	16.8	36.188	3.87	0.65	18.13	7.93

**Table A2:** **Values for diversity statistics derived from Mothur**.

Site	*z*	*S*_obs_	C1	LL	UL	ACE	LL	UL	H’	LL	UL	1/D	LL	UL	Cov
A2	18	57	111	76	209	117	92	158	3.26	3.13	3.39	16.2	13.7	19.9	0.92

A4	17	56	79	64	124	78	65	108	3.05	2.89	3.21	8.9	7.1	11.7	0.94
	43	109	216	165	316	442	362	547	3.83	3.66	4.00	18.2	14.0	25.7	0.80

A6	2	73	166	111	297	206	162	273	3.44	3.30	3.58	16.0	12.9	21.1	0.89
	20	74	116	92	171	172	136	229	3.33	3.17	3.49	11.7	9.3	15.8	0.90
	80	94	212	151	342	323	259	412	3.58	3.40	3.75	12.4	9.6	17.5	0.83
	160	76	139	104	218	198	155	262	3.42	3.25	3.58	14.4	11.5	19.2	0.87
	350	113	350	233	579	798	659	973	3.82	3.66	3.98	20.3	16.1	27.4	0.77
	700	78	181	124	304	297	228	397	3.28	3.09	3.46	10.5	8.3	14.2	0.84
	1700	130	324	234	491	520	425	645	4.05	3.90	4.21	23.7	18.6	32.8	0.77

B4	200	55	75	62	108	85	68	124	2.96	2.81	3.11	11.3	9.8	13.4	0.93
	530	75	151	110	242	332	263	427	3.25	3.08	3.42	11.4	9.2	15.0	0.86

B5	2	98	233	161	388	263	212	337	3.59	3.42	3.76	11.8	9.3	16.3	0.85
	200	57	84	67	128	143	111	194	3.21	3.08	3.34	16.0	13.6	19.3	0.92
	450	69	107	85	159	158	125	209	3.37	3.22	3.52	15.5	12.6	20.0	0.90
	760	59	144	93	274	225	170	308	2.60	2.41	2.80	4.9	4.1	6.2	0.90

C4	2	86	176	127	284	254	203	326	3.68	3.54	3.82	22.1	18.0	28.7	0.86
	200	62	100	78	154	178	139	237	2.84	2.66	3.02	6.9	5.7	8.8	0.90
	700	74	197	125	373	256	201	336	3.51	3.37	3.66	18.7	15.0	24.7	0.87

D3	25	96	202	148	314	301	241	385	3.50	3.32	3.69	10.5	8.2	14.6	0.83
	68	77	182	122	320	197	156	260	3.15	2.97	3.34	9.0	7.4	11.4	0.87

D5	2	85	122	102	168	130	108	173	3.40	3.21	3.58	8.9	7.0	12.2	0.89
	50	82	130	104	188	136	110	186	3.15	2.95	3.34	7.5	6.1	9.7	0.88
	100	78	110	92	150	129	104	178	3.05	2.84	3.26	5.9	4.7	7.7	0.89
	350	95	217	155	345	317	248	418	3.60	3.44	3.76	16.0	12.7	21.5	0.83
	450	170	719	478	1147	2101	1799	2459	4.17	4.01	4.33	21.9	17.2	30.0	0.69
	900	76	217	137	403	321	248	424	3.24	3.07	3.40	12.0	10.0	15.2	0.86

E2	6	86	167	124	259	234	187	304	3.38	3.19	3.56	9.8	7.7	13.4	0.86

E6	200	105	213	159	319	346	281	435	3.96	3.82	4.10	30.8	25.1	39.7	0.82
	800	34	46	37	73	51	40	82	1.52	1.32	1.72	2.0	1.8	2.3	0.96

F6	2	71	106	86	153	147	119	193	2.88	2.68	3.08	5.2	4.3	6.8	0.90
	200	84	152	115	232	217	174	281	3.59	3.43	3.74	17.2	13.8	23.0	0.86
	950	63	163	103	312	207	154	291	3.00	2.84	3.16	10.3	8.7	12.7	0.88

H1	7	67	94	77	136	95	80	129	3.14	2.96	3.32	8.5	6.8	11.3	0.92

H3	20	89	187	135	298	261	207	340	3.27	3.09	3.45	8.7	7.0	11.4	0.86

H6	2	58	87	69	134	120	95	162	2.77	2.59	2.95	5.7	4.6	7.3	0.92
	25	56	86	67	137	115	91	158	2.92	2.75	3.09	7.4	6.0	9.7	0.93
	45	64	87	73	124	127	102	168	3.21	3.05	3.37	10.7	8.5	14.3	0.92
	110	93	199	144	314	261	209	336	3.55	3.37	3.73	12.6	9.8	17.4	0.83
	280	84	174	124	286	226	180	292	3.70	3.57	3.84	24.9	20.7	31.1	0.86

MR1	2	83	126	103	176	192	154	250	3.64	3.49	3.80	20.3	16.3	26.9	0.86

MR2	8	91	177	133	269	263	209	342	3.44	3.26	3.62	10.6	8.3	14.5	0.84

MR3	110	101	184	142	268	174	140	237	4.02	3.87	4.17	32.1	25.2	44.1	0.80

*Sobs, observed richness; C1, Chao1; H’, Shannon index; 1/D, inverse Simpson’s index; Cov, coverage. LL and UL, 95% lower and upper confidence limits, respectively for C1, ACE, H’, and 1/D*.

## References

[B1] Agogue H. Lamy D. Neal P. R. Sogin M. L. Herndl G. J. (2011). Water mass-specificity of bacterial communities in the North Atlantic revealed by massively parallel sequencing. Mol. Ecol. 20, 258–274 10.1111/j.1365-294X.2010.04932.x 21143328 PMC3057482

[B2] Al-Awadhi H. Sulaiman R. H. Mahmoud H. M. Radwan S. S. (2007). Alkaliphilic and halophilic hydrocarbon-utilizing bacteria from Kuwaiti coasts of the Arabian Gulf. Appl. Microbiol. Biotechnol. 77, 183–186 10.1007/s00253-007-1127-1 17710391

[B3] Bano N. Hollibaugh J. T. (2002). Phylogenetic composition of bacterioplankton assemblages from the Arctic Ocean. Appl. Environ. Microbiol. 68, 505–518 10.1128/AEM.68.2.505-518.2002 11823184 PMC126663

[B4] Barberán A. Casamayor E. O. (2010). Global phylogenetic community structure and β-diversity patterns in surface bacterioplankton metacommunities. Aquat. Microb. Ecol. 59, 1–10 10.3354/ame01389

[B5] Beman J. M. Popp B. N. Francis C. A. (2008). Molecular and biogeochemical evidence for ammonia oxidation by marine Crenarchaeota in the Gulf of California. ISME J. 2, 429–411 10.1038/ismej.2008.33 18200070

[B6] Bergmann G. T. Bates S. T. Eilers K. G. Lauber C. L. Caporaso J. G. Walters W. A. (2011). The under-recognized dominance of *Verrucomicrobia* in soil bacterial communities. Soil Biol. Biochem. 43, 1450–1544 10.1016/j.soilbio.2011.03.012 22267877 PMC3260529

[B7] Berthe-Corti L. Nachtkamp M. (2010) “Bacterial communities in hydrocarbon-contaminated marine coastal environments,” in Handbook of Hydrocarbon and Lipid Microbiology, ed. Timnis K. N. (Berlin: Springer-Verlag), 2349–2359

[B8] Brown M. V. Philip G. K. Bunge J. A. Smith M. C. Bissett A. Lauro F. M. (2009). Microbial community structure in the North Pacific ocean. ISME J. 3, 1374–1386 10.1038/ismej.2009.86 19626056

[B9] Chen N. Bianchi T. S. McKee B. A. Bland J. M. (2001). Historical trends of hypoxia on the Louisiana shelf, applications of pigments and biomarkers. Org. Geochem. 32, 543–561 10.1016/S0146-6380(00)00145-5

[B10] Chin-Leo G. Benner R. (1992). Enhanced bacterioplankton production and respiration at intermediate salinities in the Mississippi River plume. Mar. Ecol. Prog. Ser. 87, 87–103 10.3354/meps087087

[B11] Church M. J. Wai B. Karl D. M. DeLong E. F. (2010). Abundances of crenarchaeal amoA genes and transcripts in the Pacific Ocean. Environ. Microbiol. 12, 679–688 10.1111/j.1462-2920.2009.02108.x 20002133 PMC2847202

[B12] Cottrell M. T. Kirchman D. L. (2000). Community composition of marine bacterioplankton determined by 16S rRNA gene clone libraries and fluorescence in situ hybridization. Appl. Environ. Microbiol. 66, 5116–5122 10.1128/AEM.66.4.1692-1697.2000 11097877 PMC92431

[B13] Dagg M. J. Ammerman J. W. Amon R. M. W. Gardner W. S. Green R. E. Lohrenz S. E. (2006). A review of water column processes influencing hypoxia in the northern Gulf of Mexico. Estuar. Coast 30, 735–752

[B14] De Corte D. Yokokawa T. Varela M. M. Agogue H. Herndl G. J. (2009). Spatial distribution of Bacteria and Archaea and amoA gene copy numbers throughout the water column of the Eastern Mediterranean Sea. ISME J. 3, 147–158 10.1038/ismej.2008.94 18818711

[B15] Dupont C. L. Rusch D. B. Yooseph S. Lombardo M.-J. Richter R. A. Valas R. (2012). Genomic insights into SAR86, an abundant and uncultivated bacterial lineage. ISME J. 6, 1186–1199 10.1038/ismej.2011.189 22170421 PMC3358033

[B16] Edwards B. R. Reddy C. M. Camilli R. Carmichael C. A. Longnecker K. Van Mooy B. A. S. (2011). Rapid microbial respiration of oil from the Deepwater Horizon spill in offshore surface waters of the Gulf of Mexico. Environ. Res. Lett. 6, 1–9 10.1088/1748-9326/6/3/035301

[B17] Eiler A. Hayakawa D. H. Rappe M. S. (2011). Non-random assembly of bacterioplankton communities in the subtropical north Pacific Ocean. Front. Microbiol. 2:140 10.3389/fmicb.2011.00140 21747815 PMC3130143

[B18] Friedline C. J. Franklin R. B. McCallister S. L. Rivera M. C. (2012). Microbial community diversity of the eastern Atlantic Ocean reveals geographic differences. Biogeosci. Discuss. 9, 109–150 10.5194/bgd-9-109-2012

[B19] Galand P. E. Potvin M. Casamayor E. O. Lovejoy C. (2010). Hydrography shapes bacterial biogeography of the deep Arctic Ocean. ISME J. 4, 564–576 10.1038/ismej.2009.134 20010630

[B20] Gerdes B. Brinkmeyer R. Dieckmann G. Helmke E. (2005). Influence of crude oil on changes of bacterial communities in Arctic sea-ice. FEMS Microbiol. Ecol. 53, 129–139 10.1016/j.femsec.2004.11.010 16329935

[B21] Giongo A. Crabb D. B. Davis-Richardson A. G. Chauliac D. Mobberley J. M. Gano K. A. (2010). PANGEA, pipeline for analysis of next generation amplicons. ISME J. 4, 852–861 10.1038/ismej.2010.16 20182525 PMC2974434

[B22] Hall B. D. Aiken G. R. Krabbenhoft D. P. Marvin-Dipasquale M. Swarzenski C. M. (2008). Wetlands as principal zones of methylmercury production in southern Louisiana and the Gulf of Mexico region. Environ. Pollut. 154, 124–134 10.1016/j.envpol.2007.12.017 18242808

[B23] Hamady M. Lozupone C. Knight R. (2010). Fast UniFrac, facilitating high-throughput phylogenetic analyses of microbial communities including analyses of pyrosequencing and PhyloChip data. ISME J. 4, 17–27 10.1038/ismej.2009.97 19710709 PMC2797552

[B24] Hazen T. C. Dubinsky E. A. DeSantis T. Z. Andersen G. L. Piceno Y. M. Singh N. (2010). Deep-sea oil plume enriches indigenous oil-degrading bacteria. Science 330, 204–208 10.1126/science.1195979 20736401

[B25] Hewson I. Steele J. A. Capone D. G. Fuhrman J. A. (2006). Temporal and spatial scales of variation in bacterioplankton assemblages of oligotrophic surface waters. Mar. Ecol. Prog. Ser. 311, 67–77 10.3354/meps311067

[B26] Hill T. C. J. Walsh K. A. Harris J. A. Moffett B. F. (2003). Using ecological diversity measures with bacterial communities. FEMS Microbiol. Ecol. 43, 1–11 10.1111/j.1574-6941.2003.tb01040.x 19719691

[B27] Hu A. Jiao N. Zhang C. L. (2011a). Community structure and function of planktonic Crenarchaeota, changes with depth in the South China Sea. Microb. Ecol. 62, 549–563 10.1007/s00248-011-9866-z 21597940

[B28] Hu A. Jiao N. Zhang R. Yang Z. (2011b). Niche partitioning of marine group I Crenarchaeota in the euphotic and upper mesopelagic zones of the East China Sea. Appl. Environ. Microbiol. 77, 7469–7478 10.1128/AEM.02402-10 21873485 PMC3209141

[B29] Jochem F. J. (2001). Morphology and DNA content of bacterioplankton in the northern Gulf of Mexico: analysis by epifluorescence microscopty and flow cytometry. Aquat. Microb. Ecol. 25, 179–194 10.3354/ame025179

[B30] Jochem F. J. (2003). Morphology and DNA content of bacterioplankton in the northern Gulf of Mexico, analysis by epifluorescence microscopy and flow cytometry. Aquat. Microb. Ecol. 25, 179–194 10.3354/ame025179

[B31] Jones K. L. Mikulski C. M. Barnhorst A. Doucette G. J. (2010). Comparative analysis of bacterioplankton assemblages from Karenia brevis bloom and non-bloom water on the west Florida shelf (Gulf of Mexico, USA) using 16S rRNA gene clone libraries. FEMS Microbiol. Ecol. 73, 468–485 20618855 10.1111/j.1574-6941.2010.00914.x

[B32] Jones R. T. Robeson M. S. Lauber C. L. Hamady M. Knight R. Fierer N. (2009). A comprehensive survey of soil acidobacterial diversity using pyrosequencing and clone library analyses. ISME J. 3, 442–453 10.1038/ismej.2008.127 19129864 PMC2997719

[B33] Kessler J. D. Valentine D. L. Redmond M. C. Du M. Chan E. W. Mendes S. D. (2011). A persistent oxygen anomaly reveals the fate of spilled methane in the deep Gulf of Mexico. Science 331, 312–315 10.1126/science.1199697 21212320

[B34] Lauro F. M. McDougald D. Thomas T. Williams T. J. Egan S. Rice S. (2009). The genomic basis of trophic strategy in marine bacteria. Proc. Natl. Acad. Sci. U.S.A. 106, 15527–15533 10.1073/pnas.0903507106 19805210 PMC2739866

[B35] Lin X. Wakeham S. G. Putnam I. F. Astor Y. M. Scranton M. I. Chistoserdov A. Y. (2006). Comparison of vertical distributions of prokaryotic assemblages in the anoxic Cariaco Basin and Black Sea by use of fluorescence in situ hybridization. Appl. Environ. Microbiol. 72, 2679–2690 10.1128/AEM.72.4.2679-2690.2006 16597973 PMC1449015

[B36] Liu B. Ye G. Wang F. Bell R. Noakes J. Short T. (2009). Community structure of Archaea in the water column above gas hydrates in the Gulf of Mexico. Geomicrobiol. J. 26, 363–369 10.1080/01490450902929290

[B37] Liu H. Dagg M. Campbell L. Urban-Rich J. (2004). Picophytoplankton and bacterioplankton in the Mississippi River plume and its adjacent waters. Estuaries 27, 147–156 10.1007/BF02803568

[B38] Liu Z. Lozupone C. Hamady M. Bushman F. D. Knight R. (2007). Short pyrosequencing reads suffice for accurate microbial community analysis. Nucleic Acids Res. 35, e120 10.1093/nar/gkm859 17881377 PMC2094085

[B39] Malmstrom R. R. Kiene R. P. Kirchman D. L. (2004). Identification and enumeration of bacteria assimilating dimethylsulfoniopropionate (DMSP) in the North Atlantic and Gulf of Mexico. Limnol. Oceanogr. 49, 597–606 10.4319/lo.2004.49.2.0597

[B40] Melcher R. J. Apitz S. E. Hemmingsen B. B. (2002). Impact of irradiation and polycyclic aromatic hydrocarbon spiking on microbial populations in marine sediment for future aging and biodegradability studies. Appl. Environ. Microbiol. 68, 2858–2868 10.1128/AEM.68.6.2858-2868.2002 12039743 PMC123915

[B41] Milkov A. V. Sassen R. (2003). Two-dimensional modeling of gas hydrate decomposition in the northwestern Gulf of Mexico, significance to global change assessment. Glob. Planet. Change 36, 31–46 10.1016/S0921-8181(02)00162-5

[B42] Olapade O. A. (2010). Molecular analyses of the diversity in marine bacterioplankton assemblages along the coastline of the northeastern Gulf of Mexico. Can. J. Microbiol. 56, 853–863 10.1139/W10-069 20962909

[B43] Pakulski J. D. Benner R. Amon R. Eadie B. Whitledge T. E. (1995). Microbial metabolism and nutrient cycling in the Mississippi River plume, evidence for nitrification at intermediate plume salinities. Mar. Ecol. Prog. Ser. 117, 207–281 10.3354/meps117207

[B44] Pester M. Schleper C. Wagner M. (2011). The Thaumarchaeota, an emerging view of their phylogeny and ecophysiology. Curr. Opin. Microbiol. 14, 300–306 10.1016/j.mib.2011.04.007 21546306 PMC3126993

[B45] Pomeroy L. R. Sheldon J. E. Sheldon J. W. M. Peters F. (1995). Limits to growth and respiration of bacterioplankton in the Gulf of Mexico. Mar. Ecol. Prog. Ser. 117, 259–268 10.3354/meps117259

[B46] Pommier T. Canbäck B. Riemann L. Bostrom K. H. Simu K. Lundberg P. (2007). Global patterns of diversity and community structure in marine bacterioplankton. Mol. Ecol. 16, 867–880 10.1111/j.1365-294X.2006.03189.x 17284217

[B47] Pontarp M. Canback B. Tunlid A. Lundberg P. (2012). Phylogenetic analysis suggests that habitat filtering is structuring marine bacterial communities across the globe. Microb. Ecol. 64, 8–17 10.1007/s00248-011-0005-7 22286378 PMC3375428

[B48] Quaiser A. Zivanovic Y. Moreira D. Lopez-Garcia P. (2011). Comparative metagenomics of bathypelagic plankton and bottom sediment from the Sea of Marmara. ISME J. 5, 285–304 10.1038/ismej.2010.113 20668488 PMC3105693

[B49] Rabalais N. N. Turner R. E. Dortch Q. Wiseman W. J. Jr. Gupta B. K. S. (1996). Nutrient changes in the Mississippi River and system responses on the adjacent continental shelf. Estuaries 19, 386–407 10.2307/1352458

[B50] Santoro A. E. Casciotti K. L. Francis C. A. (2010). Activity, abundance and diversity of nitrifying archaea and bacteria in the central California current. Environ. Microbiol. 12, 1989–2006 10.1111/j.1462-2920.2010.02205.x 20345944

[B51] Sassen R. Sweet S. T. DeFreitas D. A. Morelo J. A. Milkov A. V. (2001). Gas hydrate and crude oil from the Mississippi Fan Foldbelt, downdip Gulf of Mexico Salt Basin: significance to petroleum system. Org. Geochem. 32, 999–1008 10.1016/S0146-6380(01)00064-X

[B52] Schloss P. D. Westcott S. L. Ryabin T. Hall J. R. Hartmann M. Hollister E. B. (2009). Introducing Mothur, Open-source, platform-independent, community-supported software for describing and comparing microbial communities. Appl. Environ. Microbiol. 75, 7537–7541 10.1128/AEM.01541-09 19801464 PMC2786419

[B53] Senn D. B. Chesney E. J. Blum J. D. Bank M. S. Maage A. Shine J. P. (2010). Stable isotope (N, C, Hg) study of methylmercury sources and trophic transfer in the northern Gulf of Mexico. Environ. Sci. Technol. 44, 1630–1637 10.1021/es902361j 20104887

[B54] Stevens H. Ulloa O. (2008). Bacterial diversity in the oxygen minimum zone of the eastern tropical South Pacific. Environ. Microbiol. 10, 1244–1259 10.1111/j.1462-2920.2007.01539.x 18294206

[B55] Swan B. K. Martinez-Garcia M. Preston C. M. Sczyrba A. Woyke T. Lamy D. (2011). Potential for chemolithoautotrophy among ubiquitous bacteria lineages in the dark ocean. Science 333, 1296–1300 10.1126/science.1203690 21885783

[B56] Valentine D. L. (2007). Adaptations to energy stress dictate the ecology and evolution of the Archaea. Nat. Rev. Microbiol. 5, 36–323 10.1038/nrmicro1619 17334387

[B57] Valentine D. L. Kessler J. D. Redmond M. C. Mendes S. D. Heintz M. B. Farwell C. (2010). Propane respiration jump-starts microbial response to a deep oil spill. Science 330, 208–211 10.1126/science.1196830 20847236

[B58] Vamosi S. M. Heard S. B. Vamosi J. C. Webb C. O. (2009). Emerging patterns in the comparative analysis of phylogenetic community structure. Mol. Ecol. 18, 572–592 10.1111/j.1365-294X.2008.04001.x 19037898

[B59] Webb C. O. Ackerly D. D. McPeek M. A. Donoghue M. J. (2002). Phylogenies and community ecology. Annu. Rev. Ecol. Syst. 33, 475–505 10.1146/annurev.ecolsys.33.010802.150448

[B60] Yakimov M. M. Cono V. L. Smedile F. DeLuca T. H. Juarez S. Ciordia S. (2011). Contribution of crenarchaeal autotrophic ammonia oxidizers to the dark primary production in Tyrrhenian deep waters (Central Mediterranean Sea). ISME J. 5, 945–961 10.1038/ismej.2010.197 21209665 PMC3131861

[B61] Zinger L. Amaral-Zettler L. A. Fuhrman J. A. Horner-Devine M. C. Huse S. M. Welch D. B. (2011). Global patterns of bacterial beta-diversity in seafloor and seawater ecosystems. PLoS ONE 6:e24570 10.1371/journal.pone.0024570 21931760 PMC3169623

